# Innovative Biomaterials: The Cornerstone of Next-Generation Medical Solutions

**DOI:** 10.3390/jfb15080218

**Published:** 2024-08-02

**Authors:** Cristian Scheau, Andreea Cristiana Didilescu, Constantin Caruntu

**Affiliations:** 1 Department of Physiology, The “Carol Davila” University of Medicine and Pharmacy, 050474 Bucharest, Romania; 2 Department of Radiology and Medical Imaging, “Foisor” Clinical Hospital of Orthopaedics, Traumatology and Osteoarticular TB, 021382 Bucharest, Romania; 3 Department of Embryology, Faculty of Dentistry, The “Carol Davila” University of Medicine and Pharmacy, 050474 Bucharest, Romania; 4 Department of Dermatology, “Prof. N.C. Paulescu” National Institute of Diabetes, Nutrition and Metabolic Diseases, 011233 Bucharest, Romania

Over the past decade, 3D printing has gained traction in the medical field, and research has started to concentrate on discovering and developing new printing techniques and novel materials usable in this complex field. Initially, various typically used materials, such as plastics, metals, and elastic materials, were used to create pieces used as accessories in medical practice [1]. With time, and with increasing demand and interest in 3D-printed pieces for medical use, biocompatible materials have emerged, and subsequently, 3D printing techniques that are capable of working with these fragile and complex materials that require particular parameters, conditions, and post-processing work [2].

Many types of 3D technologies, each with specific features and limited material compatibility, are used today in a great number of industries. Still, a few stand out in terms of importance and necessity in 3D biocompatible products. Timofticiuc et al. highlighted one of the most efficient techniques, vat photopolymerization, used in creating biocompatible medical products. This is a method that holds great potential in a variety of medical sectors, such as dentistry, orthopedics, and even head and neck surgery [3].

Vat photopolymerization (such as DLP—Digital Light Projector and SLA—Stereolithography) uses UV light to polymerize various biocompatible materials such as resins, curing them in a dedicated tank. This method is used to build medical constructs that are highly compatible with the human body and carry a diminished risk of implant rejection, limiting the inflammatory response and preventing other medical complications [4,5]. In the review, a thorough classification of organic and inorganic biomaterials was proposed by the authors, where the contribution of each material in various areas of medicine was specified, offering readers a road map for known vat photopolymerization 3D-printed constructs with medical applications, such as bone grafts and scaffolds, dental implants, veneers and crowns, tissue engineering scaffolds and grafts, tracheostomy tubes, and even small intestinal villi structures [3].

However, despite the multitude of medical applications that these 3D printing techniques offer, there are limitations, and among the most relevant are the complex modifications required for the printer and materials to be capable of printing directly with viable cells [6]. In this regard, Heuer et al. specify the possibility of using a different approach to tackling the complications of 3D bioprinting, which is spraying them [7]. Burn injuries represent a significant threat for affected patients, and immediate and efficient treatment is mandatory for skin regeneration and improvement of patient outcomes. One of the earliest methods used for treating this type of injury is spraying the burn site with skin substitutes such as mixtures of viable keratinocytes and dermal-like compounds in an optimal distribution [8,9,10].

Heuer et al. tested different commercially available nozzle types to identify the ones that offer the most optimal cell distribution and viability, the highest number of cells, and the largest area of coverage, which are key elements for the effective and rapid healing of skin burns. To fulfill this purpose, four commercially available nozzles were subjected to testing with different pressures and the results were encouraging [7]. In this regard, with simple implementation, surgeons can optimize burn treatments and decrease the risk of contamination for patients with this type of injury; furthermore, cell spraying shows potential to replace skin grafts, which currently carry a high risk of rejection and infection [7].

Although cell spraying is a promising technique, further progress is necessary for the extension of the method to other medical fields, in addition to skin burn treatment. Approaching the treatment of deeper tissues requires more functionality than that offered by 3D components or surfaces sprayed with viable cells. A potential solution may be the understanding of nanoparticle (NP)-based solutions’ behavior and interaction with cells [11,12].

An interesting perspective was developed by Petcov et al. in their review on the improved efficiency of the anticancer response elicited by NP-based solutions used in oncotherapy [13]. In a combined literature review and research project on the internalization of NP-based solutions, a thorough classification of this process was developed for iron oxide NPs, where each feature such as size, shape, surface charge, hydrophobicity/hydrophilicity rigidity, and functional groups were described by the internalization mechanism for a better understanding of the variety of functions and mechanisms that NPs can use to interfere with cellular activity [13]. Once again, the importance of 3D printing was acknowledged, as an important part of understanding NP-based solutions’ effects was performed using 3D biological structures that mimic viable cells. This extensive investigation offered a better understanding of the development of drug-delivery mechanisms that can be used against tumor cells [13].

Another utility in cancer treatment was observed and researched by Park et al., where the NP mechanism of action against tumor cells follows two different directions. The authors used copper-based nanomaterials, which present increased cytotoxicity, and to overcome this disadvantage, they produced copper-loaded silica nanoparticles [14]. Based on the d-d transition mechanism of copper ions, these nanoparticles convert NIR (near-infrared) light into heat, which offers two advantages: 1. attacking tumor cells thanks to the photothermal therapy effect, and 2. bioimaging the limits of the tumor utilizing NIR fluorescence [14]. We note the opposite effects of the nanoparticles on tumor cells and the potential of this approach in oncotherapy.

As mentioned earlier, numerous 3D printing applications stand out in the dentistry realm and the great majority of these applications focus on repairing structural defects. However, the field has also expanded to cover preventive care and oral health promotion [15]. Many artificial substances may show detrimental interferences with the human body, and this has prompted research into the utility and efficiency of natural products in medicine. Thus, Budala et al. review the disadvantages of artificial products and the importance of reintroducing natural products. Oral health is a priority; it is essential for preventing bacterial-associated oral diseases, and one of the ways of addressing it is by using natural substances, such as herbal-based compounds. This is an effective method of promoting oral health, as these compounds’ secondary metabolites exhibit excellent antifungal and anticancer effects and have potential uses in periodontal, orthodontic, endodontic, restorative, and prosthetic treatments [16].

Despite its limitations, 3D printing has a promising future in fields like orthodontics and prosthodontics. Emerging methods such as cell spraying and nanoparticle constructs are gaining traction. Soon, surgeons might use adapted cell sprays for organs or tissue repair, and NP-based solutions may enhance distinct types of cell functions on top of endangering tumor cells. It is clear that the biocompatible materials described show potential in each sector addressed in this Special Issue, and future research teams must decide how these newly developed techniques using biocompatible materials will contribute to advancements in the medical field and other industries.

## Data Availability

Not applicable.
